# Surgical confidence when operating among residents in surgery – a cross-sectional study (SCAR study)

**DOI:** 10.1186/s12909-023-04389-9

**Published:** 2023-06-06

**Authors:** Alaa M. Awlia, Shomokh F. Alotaibi, Asya A. Hawsa, Abdullah O. Sultan, Nora H. Trabulsi, Nouf Y. Akeel, Nadim H. Malibary, Abdulaziz M. Saleem, Ali A. Samkari, Ahmed A. Alburakan, Mai S. Kadi, Maram T. Alkhatieb, Alaa A. Shabkah, Ali H. Farsi

**Affiliations:** 1 Department of Surgery, Faculty of Medicine, King Abdulaziz University, Jeddah, Saudi Arabia; 2 Department of Internal Medicine, King Abdulaziz Medical City, Jeddah, Saudi Arabia; 3 Department of Critical Care Medicine, King Abdulaziz Medical City, Riyadh, Saudi Arabia; 4 Department of Surgery, Dr Samir Abbas Hospital, Jeddah, Saudi Arabia; 5 Department of Surgery, College of Medicine, King Saud University, Riyadh, Saudi Arabia; 6 Department of Community Medicine, Faculty of Medicine, King Abdulaziz University, Jeddah, Saudi Arabia; 7 Department of Surgery, International Medical Center, Jeddah, Saudi Arabia

**Keywords:** General surgery residency and practice, Surgical residents and confidence, Surgical residents and trauma exposure

## Abstract

**Background:**

Self-confidence, is one of the critical variables influencing surgical resident’s abilities, and lack of confidence maybe a reason for not entering medical practice immediately. Measuring the level of confidence of senior surgical residents (SSRs) is a crucial step in assessing preparedness to practice. In this study, we aim to measure their confidence level and the factors that might contribute to it.

**Methods:**

Cross-sectional survey conducted at King Abdulaziz University Hospital on SSRs in Saudi Arabia (SA). We approached 142 SSRs, 127 responded. Statistical analysis was performed using RStudio v 3.6.2. Descriptive statistics were performed using counts and percentages for categorical variables and using mean ± standard deviation for continuous variables. Multivariate linear regression (t-statistics) was used to assess the factors associated with confidence in performing essential procedures, while the association between demographics and residency-related factor with the number of completed cases was tested using Chi-square. The level of significance was determined as 0.05.

**Results:**

Response rate was 89.4%. Among surveyed residents, 66% had completed < 750 cases as a primary surgeon. More than 90% of SSRs were confident in performing appendectomy, open inguinal hernia repair, laparoscopic cholecystectomy, and trauma laparotomy, while 88% were confident in being on-call in level-I trauma center. No difference was noted in confidence level in relation to the number of performed cases. Residents from the Ministry of Health accounted for 56.3% of the study population and showed a higher confidence level compared to others. 94% of SSRs plan to pursue fellowship training program.

**Conclusion:**

The study showed that the confidence of SSRs in performing common general surgery procedures was as expected. However, it’s important to recognize that confidence doesn’t necessarily reflect competence. Considering the majority of SSRs planned to pursue fellowship training programs, it may be time to consider changing the structure of surgical training in SA to a modular format to allow earlier and more intensive exposure.

## Background

Following graduation from medical school, doctors wishing to pursue a career in general surgery must complete the general surgery residency training. This system was first implemented by William S. Halsted at the Johns Hopkins Hospital in Baltimore, MD, USA. Prior to this, surgeons were trained by apprenticeship [1].

This was the system used since the times of the ancient Egyptians and was described in the first surgical document—the Edwin Smith Papyrus. The system continued through the centuries under the Greek physicians Hippocrates, Galen and the Arab surgeon Alzahrawi, whose book titled *Kitaab Al-Tasrif* was used as a premier surgical textbook in Europe for many centuries [2].

It was not until Halsted visited Europe, and was impressed by the formal German training, that the seeds of change were sown [1–3]. Today, surgeons are trained through formal residency training programs, and the exact details of these training programs vary from one country to another; however, in general, they adhere to Halsted’s principles of surgical training [1]. These principles are that residents must take care of patients under the supervision of a qualified surgeon, they must learn the scientific basis of surgical pathology, and they must acquire skills in patient management and surgical procedure with graded independence [4].

Today, Halsted’s principles have evolved into what can be summarized as the Canadian Medical Education Directives for Specialists (CanMEDS) framework [5]. These are the skills required of a general surgery trainee to be a competent surgeon [6] The 7 roles that a surgeon is required to play are: Medical Expert, Communicator, Collaborator, Leader, Health Advocate, Scholar, and Professional [1, 6–9].

One of the most important facets of the role of a medical expert in surgery is technical competence. This role develops during training as surgical residents are exposed gradually to more complex procedures and thereby learn to operate more competently, confidently, and efficiently. It is important to recognize here the difference between confidence and competence. Confidence refers to an individual believing that they can perform a certain skill, while competence refers to the mastery of that skill [10].

Bucholz et al. further defined self-confidence as an attitude that allows individuals to have a positive and realistic perception of themselves and their abilities [11]. It is important to recognize that surgeons who lack confidence in their skills may offer suboptimal treatment to their patients. At the same time, it can be a driver for self-improvement. As opposed to this, overconfidence can be detrimental to continued improvement and lead to skill stagnation [10].

Studies have shown that multiple factors affect confidence building. These include exposure to a variety of cases, managing critically ill patients, involvement in decision-making, operative volume, program type (university vs. hospital based vs. military), and the presence of fellows on service; all these influence the development of confidence in surgical residents [12–16]. It has been proposed that it may be possible to reduce the variation in confidence among surgical residents by standardizing their operative experience and clinical responsibilities in surgical training centers [11] however, this may not always be practical.

The importance of addressing this issue is that in an era with work hour restrictions, residents may feel that at the end of their training, they are unprepared to start medical practice on their own because of inadequate exposure to certain surgical procedures. This may lead to an elongation of the learning period by pursuing a subspecialty training program instead of directly entering surgical practice after graduation [17–19]. Subspecialty training is not always necessary unless a surgeon wishes to pursue a career with unique surgical skills that they do not normally acquire during a residency training [19].

In this study, we aimed to assess the level of confidence of senior surgical residents (SSRs) enrolled in the general surgery residency training program—overseen by the Saudi Commission for Health Specialties (SCFHS)—in performing the most prevalent procedures in general surgery and the factors associated with it. We further sought to understand their attitudes toward fellowship training, as little is known about their future career plans. In addition, we wanted to evaluate the satisfaction levels of SSRs with the different educational activities provided by their training centers, as previous research has suggested that they are dissatisfied with them [20].

## Methods

A cross-sectional study was conducted at King Abdulaziz University Hospital, during the period between October 2019–December 2020, on the SSRs of the Saudi General Surgery residency training program (a 5-year program), including 4^th^ year, 5^th^ year, and board-eligible residents who had completed training but had not yet passed their final exam. The study was approved by “The Research Committee of the Unit of Biomedical Ethics at King Abdulaziz University” (reference number 637 − 19). Signed informed consent was received from all the SSR before their enrollment in the study. All methods were carried out in accordance with relevant guidelines and regulations.

A non-probability (purposive) sampling technique was used. We approached all surgical residents attending two surgical review courses. These were 142 residents. Only 127 residents consented to participate in this survey.

The research team along with a statistician created a questionnaire based on a literature review of papers assessing the level of confidence in surgical trainees [18, 21–26]. After creating the questionnaire, it was reviewed by three academic surgeons with interest and experience in surgical education, this was followed by the distribution of the questionnaire to four general surgeons to establish face and content validity. We incorporated their feedback to modify the questionnaire. We distributed the questionnaire in a paper-based and electronic form to our target population—SSRs attending final board exam review courses in two cities.

A total of 20 multiple choice questions and 2 scale questions were used in the survey. The questionnaire asked about demographics such as age, gender, nationality, marital status, if the SSRs had children, and if they had completed their training in the city where their family lived, in addition to other questions on the type of residency and the region in which it was undertaken. We asked about their trauma experience during residency training, number of completed cases in general surgery as first surgeon at the time of answering the survey, whether they had started training directly after graduating from medical school, and if they had not, what they were involved in during the intervening period. We asked them to rank how comfortable they were with performing 25 of the most common surgical and endoscopic procedures as selected by our expert surgeons; 12 of these 25 procedures were considered essential surgical procedures and included laparoscopic colectomy, laparoscopic cholecystectomy, open inguinal hernia repair with mesh, excisional hemorrhoidectomy, modified radical mastectomy (MRM), simple mastectomy, axillary lymph node dissection, sentinel lymph node biopsy, appendectomy. We assessed if the level of the trauma centers in which the SSRs had completed their trauma rotation had any effect on their confidence in performing the three essential trauma procedures, namely splenectomy, thoracotomy, and exploratory trauma laparotomy. Plans on pursuing fellowship after residency, the chosen specialty, and the reason behind it, were all addressed in our questionnaire. Aspects of surgical practices that SSRs did not feel prepared for, were also assessed. Satisfaction with teaching activities based on the SCFHS logbook, were studied. Finally, we investigated frequency of ward rounds with consultants and whether they would have pursued a career in general surgery if the duration of the general surgery training program were 6 years instead of 5.

Statistical analysis was performed using rStudio v 3.6.2 (rStudio, PBC, Boston, MA, USA). Descriptive statistics were performed using counts and percentages for categorical variables and using mean ± standard deviation for continuous variables. With regard to the surgical and endoscopic procedures, SSRs were asked to rate their confidence in performing the procedures on a scale as follows; 1 (very confident), 2 (confident), 3 (somewhat confident), and 4 (not confident). The average confidence score was calculated for each resident twice: overall confidence and confidence in performing essential procedures. There was no previous literature mentioning either the proportion or mean confidence level among practicing surgical residents. We considered a resident’s response as confident if they selected “very confident” or “confident” for each individual procedure, while nonconfidence was defined by a resident’s selection of “somewhat confident” or “not confident.” Satisfaction with teaching activity was rated on a scale from 1 (lowest) to 5 (highest). The reliability of the confidence items (inter-item correlation) was assessed using Cronbach’s alpha. A value greater than 0.7 was considered acceptable and indicated good reliability of the scale (good average inter-item correlation).

Completed cases were dichotomized as either less than 750 or more than 750. Chi-square test and Student’s t-test were used to assess the association of demographic and residency-related factors with the number of completed cases (less than 750 or more than 750). Factors associated with confidence in performing essential trauma procedures—defined as splenectomy, thoracotomy, and exploratory trauma laparotomy—were assessed by measuring average confidence score for each participant by averaging the confidence scores for the 3 essential trauma procedures. Multivariate linear regression (t-statistics) was used to assess the factors associated with confidence in performing essential procedures. The dependent variable was defined as the average confidence in performing essential procedure (continuous). The independent variables (predictors) included age, gender, nationality, marital status, board eligibility, year of residency, residency region (province), and trauma experience level. Hypothesis testing was performed at a 5% level of significance.

## Results

### Descriptive statistics

Out of the 142 SSRs we approached, 127 responded (89.4%). Of the residents who responded, 37.8% were females. The mean age of the respondents was 30.9 ± 2.2 years. The majority of the included residents were Saudi (90.6%). More than half of the residents were married (66.1%). Slightly less than half of the included residents had children (43.3%). Approximately three-quarters of the residents practiced residency where their family lived (78.6%), as shown in Table 1. Around two-thirds of the SSRs had started their residency immediately after graduating (68.3%). The main reason for not starting a residency program immediately upon graduation was enrollment in a service job (24.41%). Other reasons included preparation for the United State Medical Licensing Exam (USMLE) (15%) and pursuing a master’s degree (10%).

**Table 1:** Descriptive statistics for the study sample

Characteristic	All Participants *N = 127*
**Gender**	
Male	79 (62.2%)
Female	48 (37.8%)
**Age*mean ± (SD***)	30.9 (2.20)
**Nationality**	
Saudi	115 (90.6%)
Other	12 (9.4%)
**Marital status**	
Married	84 (66.1%)
Single	41 (32.3%)
Divorced	2 (1.6%)
**Kids**	
Yes	56 (44.1%)
No	71 (55.9%)
**Number of kids**	
1	32 (58.2%)
2	18 (32.7%)
3	4 (7.3%)
4	1 (1.8%)
**Region of residency**	
Eastern	16 (12.6%)
Western	81 (63.8%)
Central	21 (16.5%)
Southern	9 (7.1%)
**Practicing in the same city where the family lives**	
Yes	99 (78.6%)
No	27 (21.4%)
**Residency type**	
University Medical Center	17 (13.5%)
Military Medical Center	31 (24.6%)
Ministry of Health	71 (56.3%)
Private	3 (2.4%)
Other (KFSHRC)	4 (3.2%)
**Started residency after graduating**	
Yes	86 (68.3%)
No	40 (31.7%)

The majority of the residents had completed < 750 cases as the primary surgeon in the entire residency (n = 84, 66.1%). Only 6.3% of the included residents had completed > 950 cases. Most of SSRs had been exposed to trauma cases at a Level I trauma center (55.1%), as shown in Table 2.

**Table 2:** Cases completed as the primary surgeon and trauma experience in SSR

	All Participants *N = 127*
**Number of completed cases as a first surgeon during residency**	
<750	84 (66.1%)
750–850	22 (17.3%)
851–950	13 (10.2%)
951–1150	4 (3.2%)
>1150	4 (3.2%)
**Trauma experience during residency**	
Level I Trauma center	70 (55.1%)
Level II Trauma center	30 (23.6%)
Level III Trauma center	1 (0.8%)
Outside rotation at level I	22 (17.3%)
Outside rotation at level II	3 (2.4%)
Outside rotation at level III	1 (0.8%)

### SSR attitude to fellowship

**Fig. 1:**
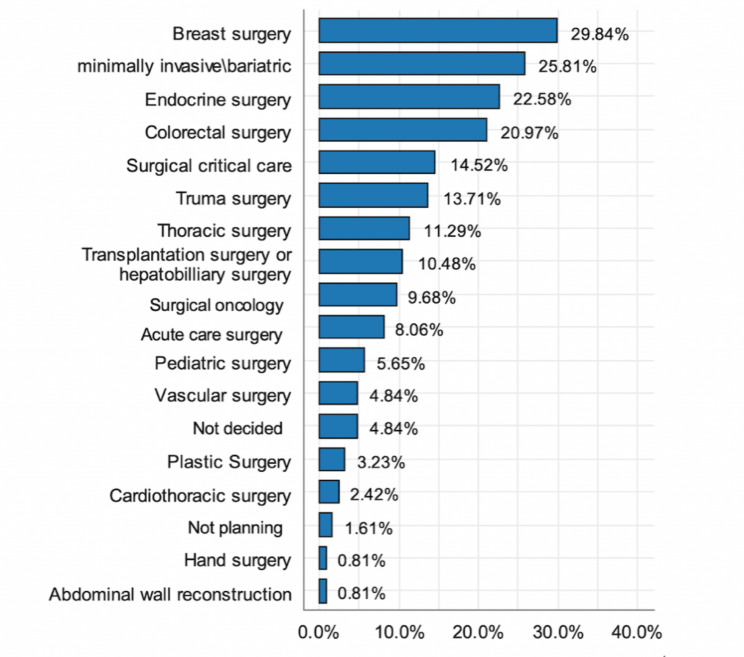
Preferred fellowship programs by SSR *Residents were able to choose more than one answer* (%yes) *(n = 124*)

Residents were asked about the fellowship programs they were planning to pursue as shown in Fig. 1. The most commonly selected fellowship programs were breast surgery and minimally invasive/bariatric and endocrine surgery; 4.8% didn’t decide yet and 1.6% of the residents were not planning to apply for a fellowship program. The main motivators for pursuing a fellowship program were educational interest (57.7%), better self-marketing (34.2%), hoping for a better lifestyle (28.5%), and planning to pursue an academic career (25.2%). Lack of confidence in surgical skills was chosen by 13.8% as another reason to pursue a fellowship program.

For those that planned on entering general practice immediately after graduation, the main factors that influenced their decision to pursue a fellowship program included a desire to pursue broad-based general surgery practice (31.5%), financial issues (27.8%), and the long duration of fellowship (29.6%). In the previously listed questions, residents were able to choose more than one answer.

Upon asking if they would have entered a general surgery residency if the training program were for a period of 6 years instead of 5, around two-thirds (64%) responded “yes.”

### Confidence in performing procedures

**Fig. 2:**
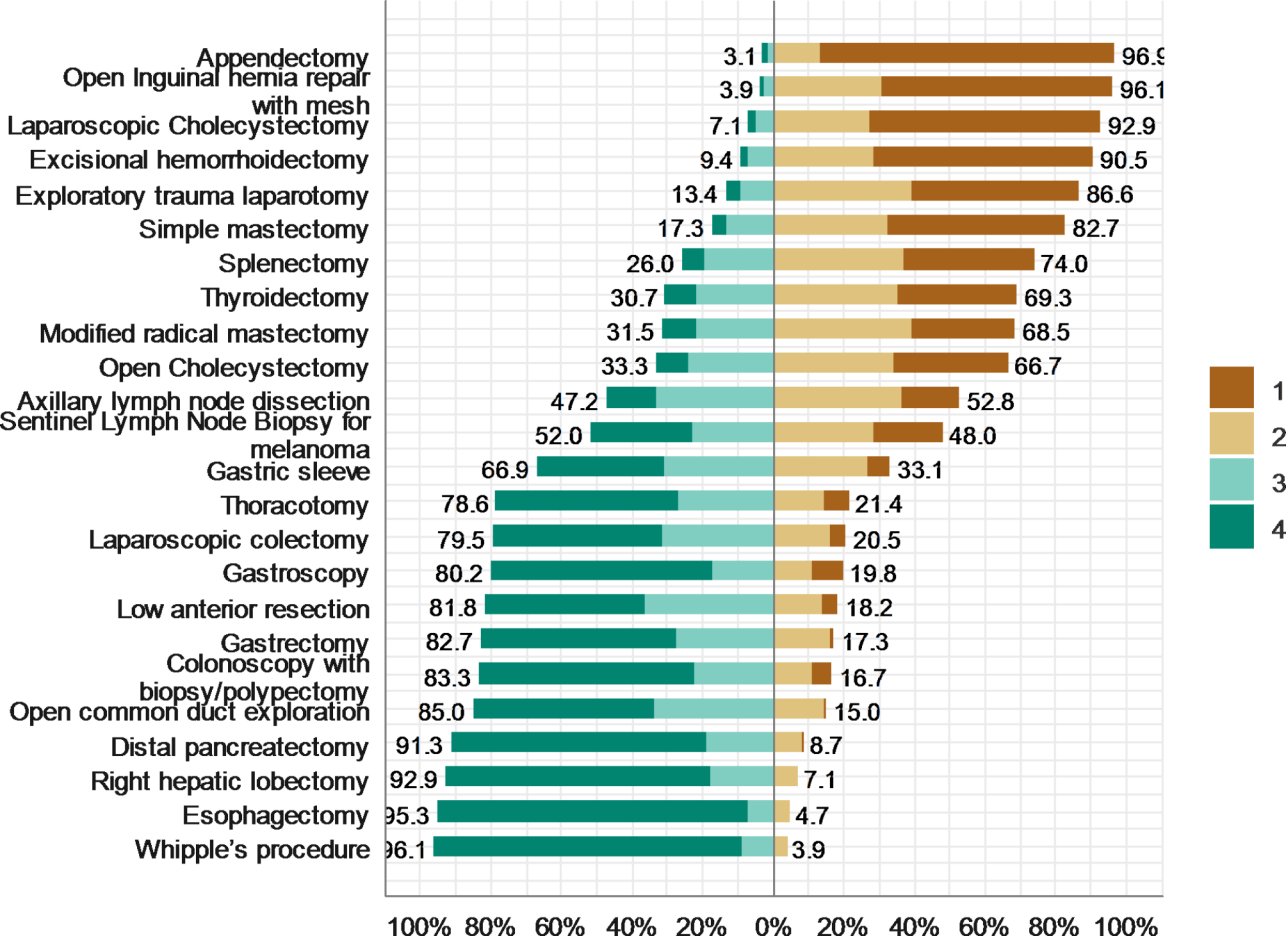
Confidence in performing the essential surgical procedures by SSR .n = 127) 1 = very confident, 2 = confident, 3 = somewhat confident, 4 = unconfident

Results showed that more than 90% of the residents were confident in performing appendectomy, open inguinal hernia repair, laparoscopic cholecystectomy, and exploratory trauma laparotomy. Residents were least confident (< 10%) in performing Whipple’s procedure, esophagectomy, right hepatic lobectomy, and distal pancreatectomy. Moreover, 88% of the residents were confident of being on call at a Level I trauma center toward the end of their residency years. The level of confidence in performing the selected procedures is shown in Fig. 2.

### Aspects of nonconfidence

**Fig. 3:**
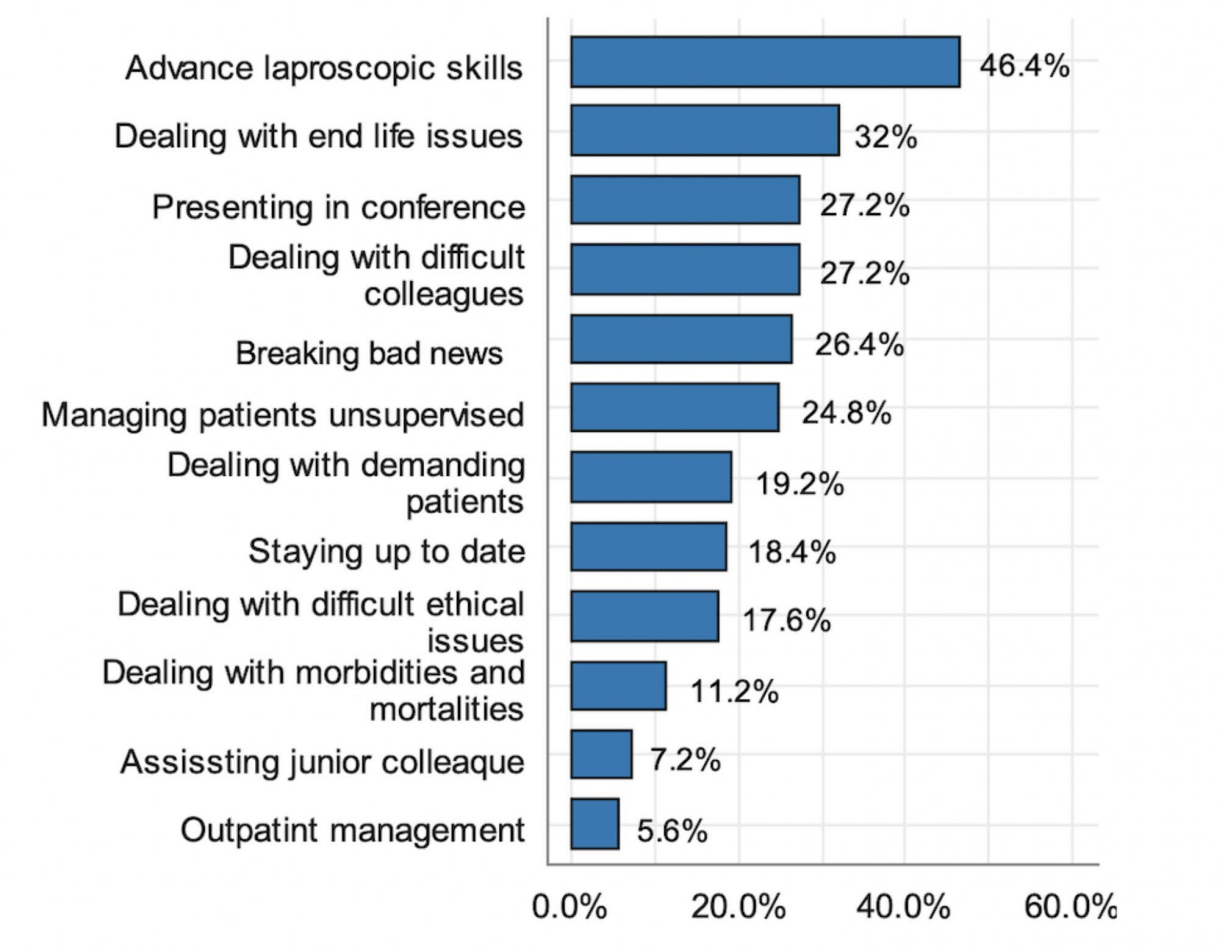
Aspects of non-confidence by SSR *Residents were able to choose more than one answer. (%yes) (n = 127*)

Areas of general surgery that SSRs believed they were not prepared to deal with independently (as perceived by them) included advanced laparoscopic skills (46.4%). Other aspects of nonconfidence are listed in Fig. 3.

### Sociodemographic characteristics and number of completed cases

With regard to factors associated with completing a higher number of cases as the primary surgeon, only increasing age was found to be significantly associated with a higher number of completed cases (P = 0.043). Other factors that were assessed are shown in Table 3.

**Table 3:** Factors associated with a higher number of completed cases

Characteristic	<750 *N = 84*	>750 * N = 43*	P-Value*
**Gender**:			*0.498*
Male	50 (59.5%)	29 (67.4%)	
Female	34 (40.5%)	14 (32.6%)	
**Age*mean ± (SD***)	30.7 (2.33)	31.5 (1.83)	0.043! *
**Residency level:**			*0.134*
Board eligible (Previous years)	12 (14.3%)	11 (25.6%)	
Board eligible (This year)	35 (41.7%)	20 (46.5%)	
Just started R5	27 (32.1%)	7 (16.3%)	
Just started R4	6 (7.14%)	1 (2.33%)	
Prefer not to mention	4 (4.76%)	4 (9.30%)	
**Nationality**:			*0.750*
Saudi	75 (89.3%)	40 (93.0%)	
Other	9 (10.7%)	3 (6.98%)	
**Marital status**:			*0.790*
Married	56 (66.7%)	28 (65.1%)	
Single	26 (31.0%)	15 (34.9%)	
Divorced	2 (2.38%)	0 (0.00%)	
**Kids**:			*0.839*
Yes	36 (42.9%)	20 (46.5%)	
No	48 (57.1%)	23 (53.5%)	
**Number of kids**:			*0.744*
1	20 (55.6%)	12 (63.2%)	
2	13 (36.1%)	5 (26.3%)	
3	2 (5.56%)	2 (10.5%)	
4	1 (2.78%)	0 (0.00%)	
**Region of residency**:			*0.360*
Eastern	12 (14.3%)	4 (9.30%)	
Western	56 (66.7%)	25 (58.1%)	
Central	11 (13.1%)	10 (23.3%)	
Southern	5 (5.95%)	4 (9.30%)	
**Practicing in the same city where the family lives**:			*1.000*
Yes	65 (78.3%)	34 (79.1%)	
No	18 (21.7%)	9 (20.9%)	
**Residency type**:			*0.212*
University Medical Center	14 (16.9%)	3 (6.98%)	
Military Medical Center	22 (26.5%)	9 (20.9%)	
MOH	42 (50.6%)	29 (67.4%)	
Private	3 (3.61%)	0 (0.00%)	
Other	2 (2.41%)	2 (4.65%)	

*Chi-square test

!Student’s t-test

### Association of completed cases with confidence in performing essential procedures

There was no statistically significant difference in the average confidence between residents based on the number of completed cases for any of the mentioned procedures with the exception of modified radical mastectomy (P = 0.009). No other statistically significant differences were observed in the mean confidence level.

### Factors associated with confidence in performing essential procedures

Multivariate linear regression analysis (t-statistics) was used to assess the predictors associated with the average confidence in performing essential procedures and demographic and residency related predictors. Positive estimates indicate a higher level of confidence. Backward elimination was used to remove the nonsignificant variables (p > 0.05) so that only statistically significant factors were retained in the final model, as listed in Table 4.

**Table 4:** Factors associated with confidence in performing essential procedures

*Predictors*	*Estimates*	*CI*	P-Value*
Intercept	3.66	1.83–5.49	*< 0.001*
Age	-0.03	-0.09–0.02	*0.221*
Gender: Male	**Ref**		
Gender = Female	0.06	-0.19–0.30	*0.655*
Level = Board eligible (Past years)	**Ref**		
Level = Board eligible (This year)	0.16	-0.12–0.44	*0.266*
Level = Just started R5	-0.03	-0.36–0.29	*0.837*
Level = Just started R4	0.27	-0.24–0.78	*0.294*
Nationality: Saudi	**Ref**		
Nationality = Other	0.33	0.01–0.66	***0.045***
Marital status = Married	**Ref**		
Marital status = Single	-0.16	-0.42–0.09	*0.201*
Marital status = Divorced	0.54	-0.18–1.27	*0.143*
Residency region = Northern	**Ref**		
Residency region = Western	0.06	-0.29–0.40	*0.747*
Residency region = Central	0.14	-0.27–0.55	*0.502*
Residency region = Southern	-0.08	-0.55–0.40	*0.757*
Residency type = Other	**Ref**		
Residency type = MOH	0.22	-0.00–0.44	***0.05***
Trauma exp = Level I Trauma center	**Ref**		
Trauma exp = Level II Trauma center	0.05	-0.19–0.29	*0.700*
Trauma exp = Level III Trauma center	0.44	-0.60–1.48	*0.404*
Trauma exp = Outside rotation at level I	0.13	-0.14–0.41	*0.345*
Trauma exp = Outside rotation at level II	-0.48	-1.09–0.13	*0.123*
Trauma exp = Outside rotation at level III	0.80	-0.19–1.79	*0.113*

*Multivariate linear regression (t-statistics)

Results showed that the mean confidence score was significantly higher in non-Saudi residents compared to Saudi residents (B = 0.33, p = 0.045). Similarly, the average confidence score was significantly higher among the Ministry of Health residents compared to other residents (B = 0.22, p = 0.05). None of the remaining demographic or residency related characteristics were significantly associated with confidence in performing essential procedures.

In our analysis, we assessed if the level of the trauma centers in which the SSRs had completed their trauma rotation had any effect on their confidence in performing the three essential trauma procedures, namely splenectomy, thoracotomy, and exploratory trauma laparotomy Results showed that there was no statistically significant association between the level of the trauma center the SSR had their trauma experience in and the average confidence in performing the abovementioned procedures (P = 0.378).

### Satisfaction with the educational activities of the sponsoring center

**Fig. 4:**
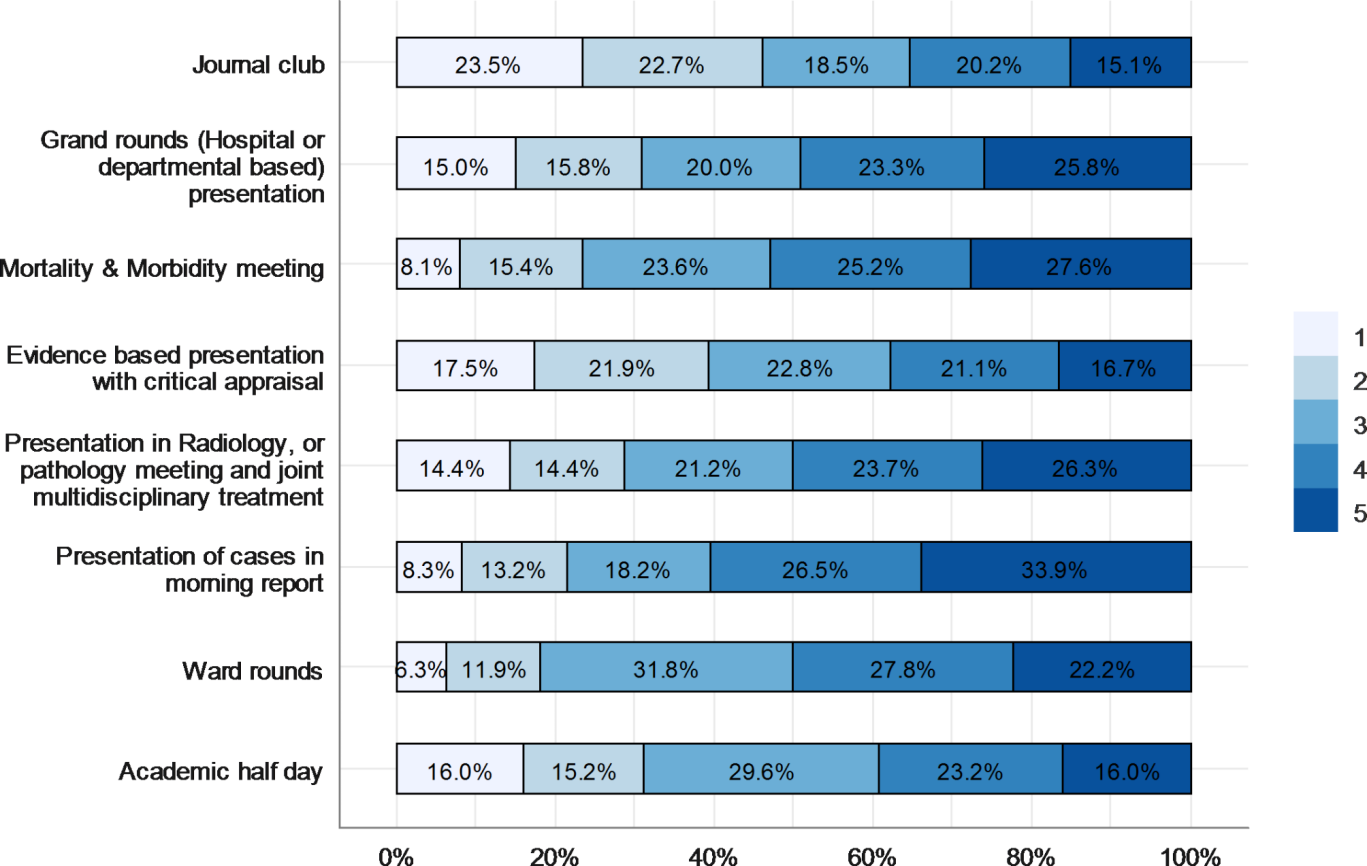
SSR Satisfaction with educational activities of sponsoring center *(5 is the highest, 1 is the lowest*)

Results showed that the most beneficial educational activity was the presentation of cases in the morning report, as stated by 33.9% of the SSRs. Further details about the residents’ satisfaction with educational activities can be found in Fig. 4. Regarding the frequency of clinical ward rounds with consultants, 82% of the residents conducted ward rounds with consultants daily, while 10%, 4%, and 3% did ward rounds twice/week, weekly, and rarely, respectively.

## Discussion

In this study, we aimed to assess the confidence of SSRs in preforming the most prevalent procedures. Confidence is gained over several years of training with the development of skills and experience. It is hard to assess the confidence level as there is no objective way to assess confidence [24].

Our first expectation was that those residents with a higher number of completed cases as primary surgeon would be more confident. Our study showed no correlation between the number of cases and the level of confidence except with regard to MRM. One explanation for the lack of significant correlation between the number of cases performed and confidence level in procedures other than MRM is the small number of residents who completed over 850 and over 950 cases as the primary surgeon. In previous studies, evidence is conflicting regarding whether the number of completed cases increases a resident’s confidence in the operation room or not. Some studies have found an association [24, 27, 28], while others have not [18, 23, 25]. The authors who found no difference have suggested that after completing a certain minimum number of cases, surgical resident’s confidence plateaus [25]. It would also seem that building confidence among surgical residents is a multistep process and does not rely solely on caseload [10]. Previous research that assessed what elements residents (both medical and surgical) identify as confidence building include working through decisions with the consultant, managing sick patients, having good support from other residents, being given responsibility for critical issues in patient management, and making decisions on their own, among many other factors [12]. Jason et al. evaluated surgical residents safety in performing common procedures along with other non-technical skills, in terms of decision making, leadership, situational awareness, communication and teamwork, via an online evaluation questionnaire by the attending surgeon [29].

We believe the correlation found in MRM procedure, could be explained by the fact that breast cancer is the most common malignancy among females in Saudi Arabia (SA), [30] and MRM is a very common procedure performed in almost every hospital regardless of its facilities. This theory is further supported by the finding that breast surgery was the most commonly selected fellowship among our respondents.

With regard to predictors of increased confidence, we found that SSRs sponsored by the Ministry of Health hospitals had a higher confidence level compared to their peers. This could be related to the fact that the Ministry of Health hospitals usually have a larger variety of cases due to wider eligibility criteria for patient admission. Non-Saudis were also found to be more confident in performing the aforementioned surgical procedures. This might be because entry into the program is more competitive for them due to the limited number of seats.

We found no statistically significant difference in the level of SSR confidence based on gender. Other research has shown male gender to be predictive of increased confidence [11, 17, 24, 27]. Other papers studied external factors affecting resident confidence, such as a healthy supportive work environment, and internal factors, in terms of resident self-expectations, stress and mental health [31].

It has also been shown that when asked to assess themselves in the operating room, general surgery residents tend to underestimate their abilities compared to the supervising surgeons’ assessment, with females tending to underestimate themselves to a greater extent than their male colleagues (though this was not statistically significant) [32]. This finding is not limited to surgery, as it has been demonstrated even outside the medical field. It has been shown that when females perform a task well they are likely to attribute it to their good fortune, whereas, if it is performed poorly, they attribute it to a lack of skill, with the opposite being true in males [33].

Older age was the only factor associated with a higher number of completed cases during the residency. Regardless of the number of completed cases, the confidence level was not altered.

Nearly 94% of the SSRs in this study plan to pursue a fellowship program. The range quoted in the literature we reviewed showed this percentage to be between 60 and 80% in North America [22, 23, 25, 34]. Another study showed that the number of surgeons in the USA pursuing fellowship is increasing: from 60% to 1989 to 80% in 2011 [19]. The reasons the SSRs showed such a large interest in pursuing fellowship could be multifactorial. Reasons could include SSRs feeling that the general surgery field in SA is very competitive. That they need to have subspecialty training, in order to get a good job in a prestigious hospital or a large city. Additionally, residents suppose that they still need more operative exposure to develop their skills. Coleman et al. suggested that fellowships offer more operative experience which overcomes the confidence issues that many residents have faced [22].

In our study, we found the main motivator for fellowship training was a strong interest in the specialty, with only 13.8% stating that the lack of confidence was one of the reasons for pursuing a fellowship. This large percentage of residents wishing to pursue fellowship may support the idea of a modular format of surgical training. This has been advocated by some surgical educators in North America. In this case, a basic surgical core curriculum is introduced for the first 2–3 years of surgical training, followed by one of two pathways, a specialist in surgery (such as hepatobiliary, thoracic, colorectal, etc.) or general surgery training [35]. Previous research shows that many residents in the USA support this [22]. A potential downside could be a shortage of “general” surgeons. It could also contribute to the graduation of surgeons who are not needed. A modular format would allow subspecialty certification only, while the current standard allows certification in two specialties, namely general surgery, and the subspecialty [36].

The most selected fellowship program among the SSRs in this study was breast surgery, followed by minimally invasive/bariatric surgery. This might be because both of these fellowships deal with very common surgical conditions, with a definite need in many institutions and hospitals.

Trauma is considered one of the major areas of general surgeons’ practice; therefore, measuring the surgical residents’ preparedness in this field is critical. Previous research has shown that the volume of trauma the SSRs are exposed to has been decreasing [37]. Considering this, we enquired about the levels of trauma centers in which the SSRs received their trauma training. We expected that training in Level I trauma centers would be reflected on the confidence level; however, this was not what we found. In fact, there was no difference between the level of the trauma center and the level of SSR confidence in performing essential trauma procedures. This might be because most residents surveyed had their trauma rotation in Level I trauma centers.

The main limitation of this study is that the main outcome of interest was confidence and not actual competence. Confidence is a self-reported measure that may be biased by many factors, such as one’s sense of self-belief, insight, and fear of judgement by peers, despite a survey’s anonymity. Though the level of confidence in the procedures did match our expectation overall, there is disagreement in the literature as to what is an acceptable level of confidence. What is the minimum acceptable percentage of SSRs who should be comfortable performing a laparoscopic cholecystectomy or colectomy? There were SSRs in our study who rated themselves as not confident in performing appendectomies, for example. We also did not assess the SSRs’ abilities in other areas such as clinical decision making outside the operating room. The sample size was not large, and that could have affected detecting significant difference across the different subgroups. The sample could have been biased with more confident residents responding because it was distributed at a review course, which would have been attended by more prepared SSRs. Most respondents were from large cities, which may have influenced some of the answers such as the plans for fellowship training. However, our study provides educators with some evidence for the areas of weakness in SSR training. It helps administrators plan subspecialty training needs. We had a high response rate of over 89%. We suggest the next step should be a study correlating resident confidence and competence in the operating room with the assessment of an unbiased experienced surgeon to gauge their ability to assess themselves, while at the same time identifying areas of discrepancy between them and their educator’s assessment.

## Conclusion

Our study showed that the surgical resident’s the level of confidence in performing common general surgery procedures is appropriate for what would be expected. However, it is important to recognize that confidence does not reflect competence, though it is an important aspect of surgical training. The majority of residents felt that they would be comfortable managing trauma cases after graduation. Considering that most of the SSRs planned to continue in a fellowship training program after graduation, we suggest it may be time to consider changing surgical training in SA to a modular format that allows earlier and more intensive exposure to the residents’ area of interest.

## Data Availability

The datasets used and/or analyzed during the current study are available from the corresponding author on reasonable request.

## List of Abbreviations

CanMEDS: Canadian Medical Education Directives for Specialists
MRM: Modified Radical Mastectomy
SA: Saudi Arabia
SCFHS: Saudi Commission for Health Specialties
SSRs: Senior Surgical Residents
USMLE: United State Medical Licensing Exam
